# Aripiprazole-Induced Orofacial Dyskinesia in a Young Male: A Case Report

**DOI:** 10.7759/cureus.73269

**Published:** 2024-11-08

**Authors:** Udai Godhania, Jayaprakash Rajendran, Oluwagbenga Odeyemi

**Affiliations:** 1 Psychiatry, Priory Hospital, Birmingham, GBR

**Keywords:** aripiprazole, bipolar affective disorder, epse, extrapyramidal side effects, orofacial dyskinesia, recurrent psychosis, #tardive dyskinesia

## Abstract

We report the case of a 23-year-old man who developed orofacial dyskinesia secondary to aripiprazole whilst being treated for psychosis in the hospital. He was known to mental health services and had suffered a relapse of bipolar affective disorder. Upon cessation of aripiprazole and commencement of quetiapine, there was a rapid reversal of his movement disorder. He also did not have any notable risk factors associated with developing orofacial dyskinesia beyond having an affective disorder, which highlights the unusual and rare occurrence of this side effect with the use of aripiprazole. Unlike many first-generation antipsychotics, aripiprazole had been previously reported to be protective against and also treat extrapyramidal side effects (EPSE) due to its mixed mechanism of action, which includes being a partial dopamine receptor 2 (DR2) agonist.

## Introduction

Orofacial dyskinesia, or tardive dyskinesia, is noted by rhythmic abnormal involuntary movements of the face, mouth, tongue, trunk, and limbs. It is mostly associated with first-generation psychotropic medication. In certain rare instances, the choreiform movements can become a permanent feature, even upon cessation of the causative medication. There is also a significant amount of social stigma associated with this movement disorder that can complicate a recovery further [1].

Most cases of orofacial dyskinesia occur in combination with blepharospasm or dystonia in other areas of the body, and focal dyskinesia only accounts for 2-23% of the cases. In our case, our patient only experienced focal dyskinesia, which accounts for a minority of cases globally [2].

Aripiprazole is a second-generation antipsychotic, also referred to as an atypical antipsychotic. This latest second generation also includes other psychotropic medications such as cariprazine, brexpiprazole, and lurasidone. They have been shown to have a reduced propensity for extrapyramidal symptoms (EPS) and enhanced efficacy in improving cognitive and negative symptoms. They are also less likely to cause metabolic dysfunction. Aripiprazole has shown efficacy in the treatment of schizophrenia and acute manic episodes associated with bipolar affective disorder. Its mechanism of action allows stabilization of dopamine (DA) and serotonin (5-HT) activity within the nucleus accumbens (NAc), ventral tegmental area (VTA), and frontal cortex (FC), which explains its holistic ability to manage both negative, positive, and cognitive symptoms [3].

Aripiprazole is a partial agonist at serotonin 5-HT1A and dopamine D2 receptors and an antagonist at the serotonin 5-HT2A receptor. However, it has been reported in the literature that it has a broader pharmacological role at the dopamine D2 receptor. Depending on endogenous dopamine levels and signaling status, aripiprazole may act as a full antagonist, a moderate antagonist, or a partial agonist at dopamine D2 receptors [3].

It was previously acknowledged that aripiprazole is a consistent partial DR2 agonist, but some studies have shown it is a functionally selective DR2 ligand. When there is excess DA transmission, it can behave as a functional antagonist, and with reduced DA transmission, it acts as a functional agonist. It has shown affinity to both presynaptic and postsynaptic DR2 receptors in the mesolimbic and mesocortical pathways. This may account for its clinical action in causing side effects such as oral facial dyskinesia under certain conditions and acting essentially as a first-generation antipsychotic in blocking striatal DR2 receptors [3,4,5]. In this case report, we illustrate a case of aripiprazole-induced orofacial dyskinesia in a patient who also did not have many clinical risk factors associated with the development of this movement disorder.

## Case presentation

On April 20, 2024, a 23-year-old patient of South Asian origin was admitted to a psychiatric intensive care unit due to psychosis and a relapse of bipolar affective disorder. He had presented with increased energy, elevated mood, agitation, rapid speech, grandiosity, impaired sleep, and increased risky behavior that was leading to social and functional complications.

In the community, he had been compliant on aripiprazole, and it was effective in managing his condition. However, it was causing a mild mandibular and facial movement disorder with secondary speech impediment. Due to his relapse and severe agitated presentation, he was initially prescribed zuclopenthixol dihydrochloride 10 mg three times a day. This medication was utilized only for six days and did not result in a deterioration of his movement disorder. Additionally, it has a half-life of 20 hours (range 12-28 hours) and is thus completely eliminated from the body within two days [6].

Subsequently, he was prescribed aripiprazole intramuscular depot 200 mg with supplementary oral tablets, which resulted in a marked increase in his oral facial dyskinesia. It is noted that aripiprazole also has a longer half-life at approximately 75 hours. It was a monthly depot and 50% of the British National Formulary (BNF) maximum. He was additionally prescribed lamotrigine 300 mg twice a day as a mood stabilizer. During this period of time, he was unable to effectively communicate and was observed making abnormal movements limited to the mouth, face, jaw, and tongue. This includes grimacing, pursing of the mouth and lips, and writhing of the tongue. His mouth would also remain open intermittently during speech for several seconds due to stiffness. These movements had a secondary impact and altered the articulation of his speech as well as affecting his ability to chew and swallow food. He was prescribed clonazepam 1 mg three times a day and vitamin E, which did not result in a clinically significant improvement concerning his movement disorder. Vitamin E and clonazepam have been reported to alleviate tardive dyskinesia-like presentations. He also used procyclidine 2.5 mg three times a day as a precautionary measure against other extrapyramidal side effects (EPSE); however, he did not experience these symptoms. There was no akathisia or Parkinsonism such as a resting tremor, general rigidity, or bradykinesia. He also did not have chorea or athetosis.

Routine blood tests showed no acute abnormalities concerning his full blood count, liver and thyroid function tests, as well as urea and electrolytes. He had no history of any physical health conditions and was an avid fitness enthusiast. A general clinical examination showed no other neurological deficits or any other signs and symptoms.

**Figure 1:**
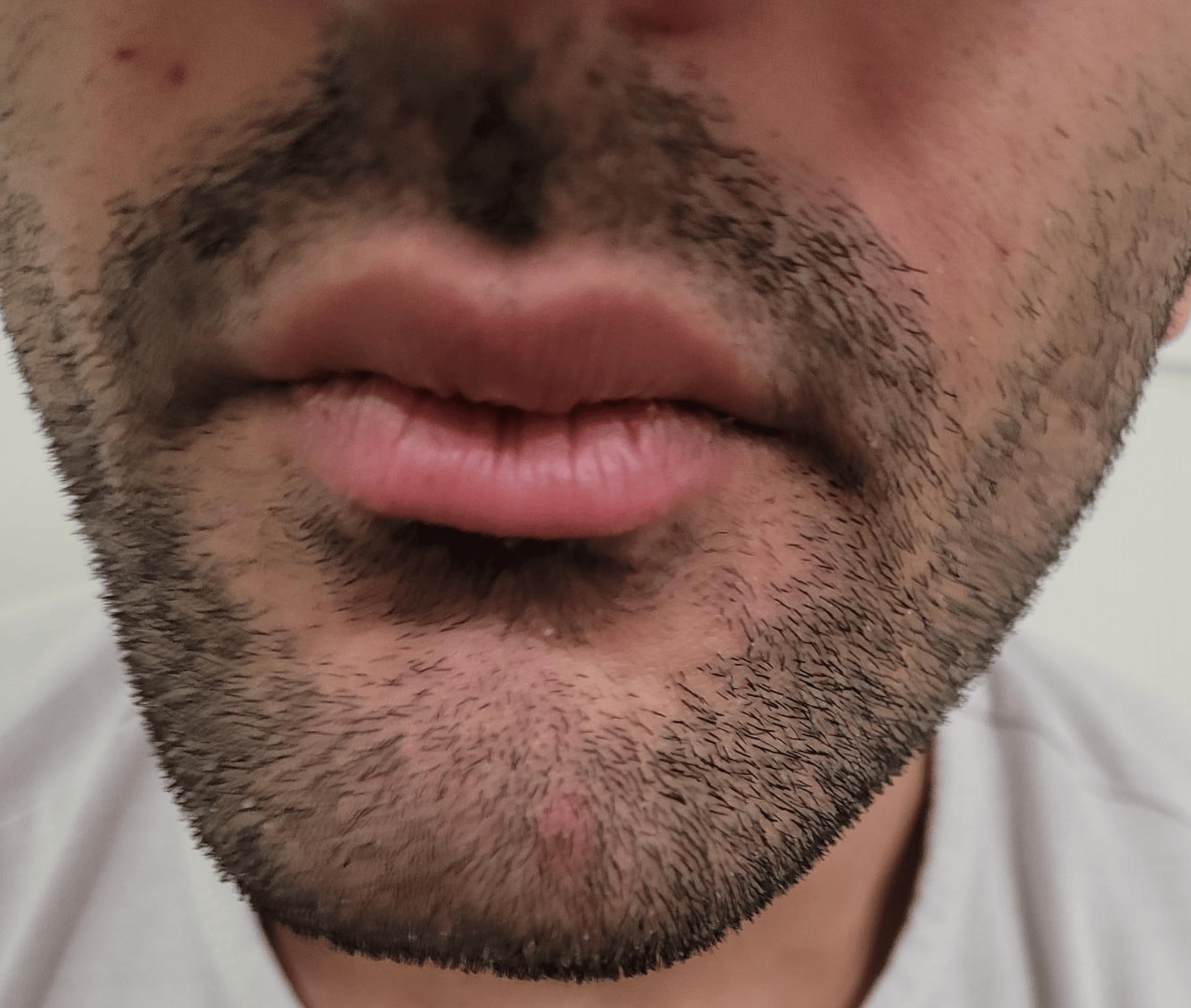
Inner cheek biting and pursing of lips during initiation of speech.

**Figure 2:**
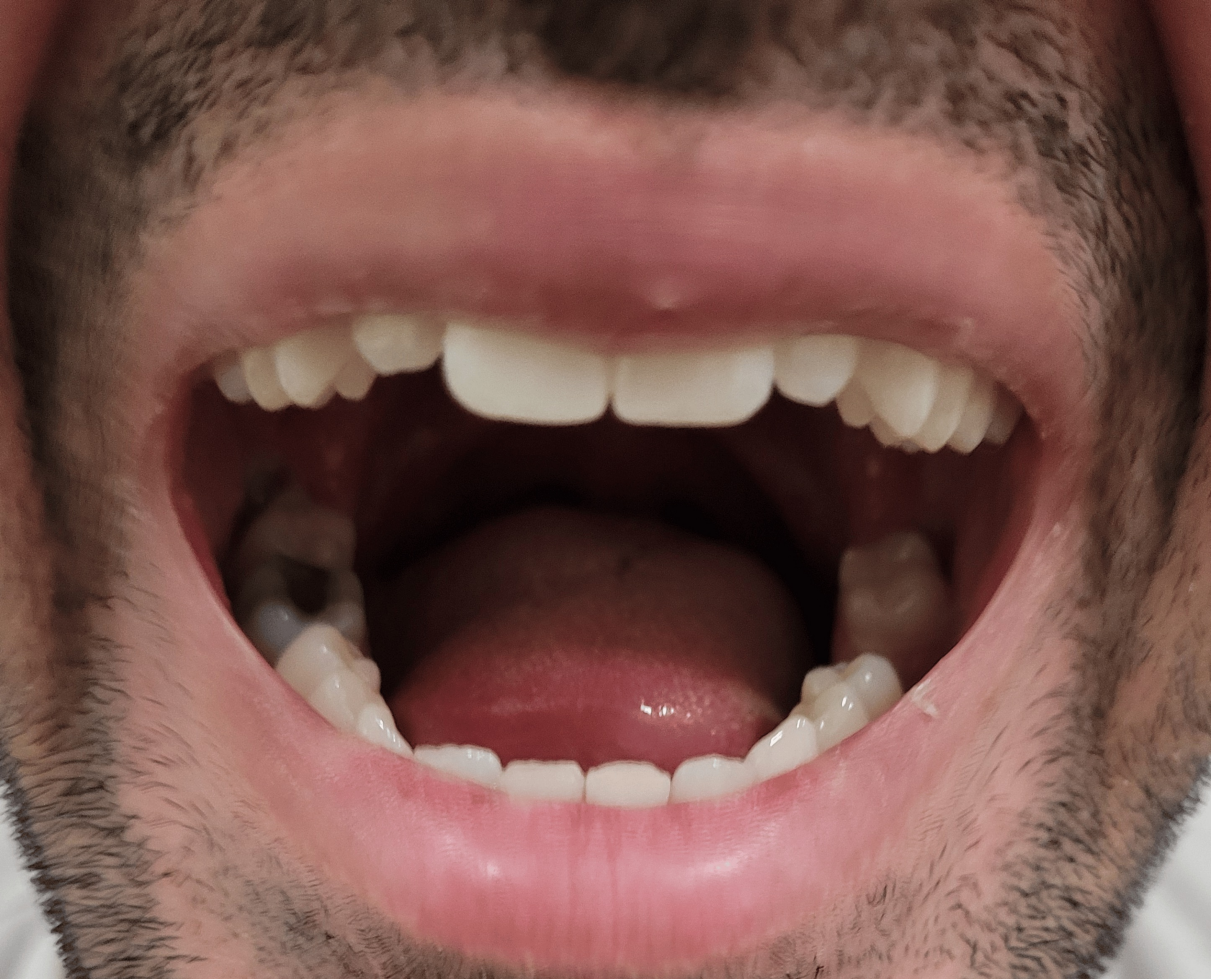
Mouth remaining open intermittently during speech for several seconds.

The patient himself had described developing a sensory routine to communicate more effectively, which included initially biting the inside of his cheek, followed by pursing of his mouth and lips, and then rolling his tongue inside his mouth. He would be observed at times with facial grimacing and tongue protrusion. He stated this allowed him to overcome his dyskinetic movements to an extent and improve his speech when communicating with other patients and staff. He was warned about the risk of an ulcer and infection due to biting the inside of his cheek. Occasionally the dyskinesia was so profound that his mouth would remain open for several seconds. Studies have highlighted secondary oral health issues that may result from orofacial dyskinesia and the need for prevention and treatment [2]. Figures 1, 2 show our patient displaying characteristic abnormal movements of orofacial dyskinesia during speech.

After several weeks, he was prescribed quetiapine 400 mg twice a day and Depakote titration up to 1 g twice a day. On examination, there was a dramatic shift in his oral facial movements towards normalization, and subjectively, he felt he had returned to his premorbid level of speech and ability to eat. He no longer needed to attempt his old routine of bizarre facial movements to combat dyskinesia. The Abnormal Involuntary Movement Scale (AIMS) showed a reduction from 20 to 2.

Clinically, the acute psychosis was heading towards remission with both regimens; however, it was vital to restore his normal mouth movements, speech, and swallowing reflex to premorbid status. It was also affecting his self-esteem as he was becoming self-conscious, and this could impede a social and functional recovery. The patient is a high-achieving professional who wished to restore his health as soon as possible so he could return to his pre-morbid level of functioning.

## Discussion

The incidence of orofacial dyskinesia with second-generation antipsychotics was estimated to be 0.8% in non-elderly adults, compared to 5.4% with first-generation antipsychotics [7]. Aripiprazole had an incidence of 0.9% in a study that involved 1215 subjects [7].

The present case demonstrates that aripiprazole can (in less than 72 hours) induce focal orofacial dyskinesia, despite it being noted overall as a rare side effect. In our case, our patient also did not have additional risk factors for developing dyskinesia aside from having an affective disorder. Additional risk factors include being antipsychotic naive, female gender, older age, diabetes mellitus, dose and duration of psychotropic therapy, intellectual disability, brain injuries, smoking, alcohol, substance misuse, and Caucasian or African ethnicity. Our patient was 23 years old, male, physically healthy, a high-achieving professional, of Asian ethnicity, and had utilized antipsychotic medication for the past two years. He also did not develop Parkinsonism and was actively playing football and going to the gym within the hospital setting. He also did not smoke or drink alcohol [8].

A case report, in keeping with some earlier studies, has highlighted a rare but possible complication that switching to aripiprazole treatment can improve tardive dyskinesia [9]. We would urge caution before attempting aripiprazole therapy for the dual purpose of improving tardive dyskinesia and managing psychosis.

There is a long-standing view that aripiprazole has the same efficacy as typical and other atypical antipsychotics but a better safety profile. This was demonstrated in a 2017 literature overview, which included systematic reviews with meta-analyses [10]. This case highlights that clinicians must carefully weigh up the benefits and risks of aripiprazole for each patient to prevent unwanted complications and potential delays in their overall recovery. Orofacial dyskinesia can lead to social stigma and potentially be irreversible [11,12].

In contrast to this, quetiapine has been demonstrated in a study to show early and long-lasting results in the treatment of dyskinetic symptoms in the orolingual region. In one case, a patient who had switched to quetiapine due to dyskinesia had an improvement from 19 to 3 on AIMS. The dose was 600 mg, and our patient was prescribed 800 mg and achieved a better reduction with AIMS [13]. This may be suggestive of a dose-dependent relationship, but further studies would be needed to determine a causal link.

Both quetiapine and clozapine are safer options than aripiprazole in minimizing the risk of orofacial dyskinesia due to their reduced dopamine receptor occupancy. Aripiprazole can occupy striatal DR2 receptors above 90%, dependent on the dose. It is a dopamine agonist in hypodopaminergic states, while it is a dopamine antagonist in hyperdopaminergic states. Aripiprazole was also more likely to antagonize postsynaptic DR2 receptors and function as an agonist at presynaptic DR2 receptors. This peculiar, mixed, and clinically unpredictable action suggests that quetiapine and clozapine have a better safety profile in avoiding dyskinetic dysfunction [3,13-16].

## Conclusions

In conclusion, this is a case in which aripiprazole induced acute and severe orofacial dyskinesia in a patient who clinically appeared to have a low propensity to develop it. In the literature, aripiprazole remains a gold standard treatment for first-episode psychosis due to its safety profile and efficacy. However, this needs to be balanced against concerns of inducing facial dyskinetic disorders, which potentially have a poor prognosis and can be irreversible. In the short term, it can lead to delayed discharge from the hospital, but in the long term, speech, eating, and drinking challenges may persist, requiring further assessment. This case highlights the importance of judicious prescribing, objective monitoring of involuntary movements, prompt withdrawal of the causative medication, and rapid reversal of this EPS with an alternative antipsychotic. Quetiapine successfully treated and reversed orofacial dyskinesia whilst ensuring there is no acute relapse in a patient with bipolar affective disorder. 
